# MDM2/Notch-Ferroptosis crosstalk in cancer: metabolic rewiring, immune evasion, and organ-specific metastasis

**DOI:** 10.7717/peerj.21480

**Published:** 2026-06-25

**Authors:** Peng Lu, Lei Zhang, Jing-Jing Fan, Yuezhen An, Lan Chen, Xiaoyun Dong, Jingxiang Ma, Yan Sun

**Affiliations:** 1 Department of Clinical Laboratory, Cangzhou Central Hospital, Cangzhou City, Hebei, China; 2 Department of Emergency ICU, Cangzhou Central Hospital, Cangzhou City, Hebei, China

**Keywords:** MDM2 and Notch signaling, Ferroptosis regulation, Metabolic reprogramming, Tumor immune microenvironment, Organotropic metastasis, Lipid peroxidation

## Abstract

Ferroptosis, an iron-dependent form of regulated cell death driven by lipid peroxidation, is increasingly recognized as a contributor to tumor progression and therapy response. Two of the most widely studied oncogenic regulators, the E3 ubiquitin ligase MDM2 and the Notch receptor family, have been independently reported to modulate ferroptosis through effects on p53/SLC7A11, NUMB/Notch/GPX4, and mitochondrial metabolism. This review surveys the primary experimental evidence for these interactions and evaluates the extent to which MDM2- and Notch-dependent pathways converge on ferroptosis effectors across cancer types. We then examine how the resulting metabolic changes, mitochondrial respiration, lipid peroxidation, cystine/glutathione homeostasis, and iron handling, intersect with tumor immune evasion and with organ-specific metastatic colonization. Throughout, we distinguish mechanistically coupled interactions (demonstrated in a single experimental system) from parallel or correlative observations across disparate systems. We conclude by outlining specific experimental gaps, including the scarcity of matched MDM2 and Notch manipulation data in single-tumor models, the unresolved context dependence of Notch’s pro- and anti-ferroptotic effects, the dual role of ferroptosis in anti-tumor immunity *versus* immune-cell fitness, and the lack of validated clinical biomarkers. The synthesis is intended as a resource for investigators designing mechanistic studies, rather than as a prescriptive therapeutic framework.

## Introduction

Ferroptosis is an iron-dependent form of regulated cell death characterized by the lethal accumulation of lipid hydroperoxides on cellular membranes (Dos Santos et al., 2023). Its biochemical core involves three interacting layers. First, labile ferrous iron (Fe2+) catalyzes Fenton chemistry that generates hydroxyl radicals from hydrogen peroxide, initiating lipid-radical cascades. Second, polyunsaturated fatty acids (PUFAs) are esterified into membrane phospholipids by ACSL4 and LPCAT3 and are subsequently oxidized by lipoxygenases (*e.g.*, ALOX15) to produce lipid hydroperoxides (Lin et al., 2025a). Third, two principal defense systems counter this oxidative attack: the glutathione peroxidase GPX4, which uses reduced glutathione (GSH) to detoxify lipid hydroperoxides, and the CoQ10/FSP1 axis, which regenerates antioxidant radicals at the membrane. GSH levels in turn depend on cystine uptake *via* the System xc^−^ antiporter, whose functional subunit is SLC7A11. Canonical experimental inducers of ferroptosis include erastin (SLC7A11 inhibitor) and RSL3 (GPX4 inhibitor); ferrostatin-1 and liproxstatin-1 are lipophilic radical-trapping antioxidants used to confirm the ferroptotic nature of a cell-death phenotype (Wang et al., 2021; Chen et al., 2022a; Greenough et al., 2022; Liu et al., 2022a; Liu et al., 2022b; Akiyama et al., 2023).

Against this biochemical backdrop, two oncogenic signaling systems have been repeatedly implicated in ferroptosis regulation. Mouse Double Minute 2 homolog (MDM2) is an E3 ubiquitin ligase best known for ubiquitinating p53 and targeting it for proteasomal degradation; MDM2 also acts on several non-p53 substrates relevant to ferroptosis, including NUMB and AGPS (Wang et al., 2021; Haronikova et al., 2021; Xu et al., 2021). The Notch family comprises four single-pass transmembrane receptors (Notch1–4). On ligand engagement, γ-secretase releases the Notch intracellular domain (NICD), which translocates to the nucleus and activates RBP-J*κ*/CBF1-dependent transcription of Hes/Hey and other target genes. In cancer, Notch signaling influences stemness, differentiation state, and the composition of the tumor microenvironment (TME) (Chen et al., 2022a; Chen et al., 2022b; Eirew et al., 2022). Ferroptosis regulation can therefore be affected by MDM2 or Notch at multiple levels: direct ubiquitination of ferroptosis effectors (*e.g.*, AGPS Zhang et al., 2024), p53-dependent transcriptional control of SLC7A11 (Rodriguez, Schreiber & Conrad, 2022; Zhang et al., 2022), and NICD-mediated transcriptional or direct protein-level effects on GPX4 (Han et al., 2023; Hu, Zhang & Zhao, 2023; Jin et al., 2024).

Although the individual components of these pathways are well characterized, reports linking MDM2 and Notch jointly to ferroptosis come from heterogeneous systems (cardiomyocytes, renal tubular cells, colorectal and liposarcoma cell lines, *etc.*) and rarely perturb both regulators within one experimental model. This review therefore has three aims: (i) to summarize the primary evidence for MDM2-ferroptosis and Notch-ferroptosis interactions and to make explicit which interactions have been demonstrated in a single system *versus* inferred from parallel observations; (ii) to examine how the metabolic consequences of these interactions intersect with tumor immune evasion and organ-specific metastasis; and (iii) to identify unresolved questions and experimental priorities. We make no claim that MDM2 and Notch constitute a formally validated co-regulatory module, and we distinguish throughout between directly demonstrated coupling, convergent but independently reported findings, and speculative integration.

### Reported interactions between MDM2/Notch signaling and ferroptosis

The direct link between MDM2 and ferroptosis involves MDM2-mediated ubiquitination and degradation of target proteins (*e.g.*, AGPS or p53), which modulate ferroptosis sensitivity. In prostate cancer cells, MDM2-dependent degradation of AGPS promotes ferroptosis (Kim et al., 2023b), whereas in non-small cell lung cancer models, MDM2 silencing stabilizes p53, downregulates SLC7A11, and induces ferroptosis (Lee & Roh, 2023; Tien et al., 2023). Conversely, the Notch pathway often antagonizes ferroptosis by promoting stemness and differentiation. In a colorectal cancer model infected with *Fusobacterium nucleatum*, MDM2-mediated Numb degradation activates Notch signaling, enhancing stem-like properties and conferring ferroptosis resistance through lipid droplet accumulation (Zhang et al., 2023b), this is one of the few studies in which MDM2-dependent NUMB degradation, Notch activation and ferroptosis readouts are all measured in a single cancer system, and we therefore classify it as “mechanistically coupled”. Paradoxically, Notch activation can also reduce mitochondrial respiration and increase ferroptosis susceptibility, as reported in dedifferentiated liposarcoma (DDLPS) cell lines *via* Notch-mediated repression of PGC-1α (Zhang et al., 2023c). A direct protein-level interaction has been described in which Notch1 binds to and stabilizes the ferroptosis suppressor GPX4 through its RAM domain (Cai et al., 2024; Cao et al., 2024) in cardiomyocyte and tumor contexts, an interaction we also classify as mechanistically coupled. These apparently opposite effects of Notch on ferroptosis are reconciled in ‘Reconciling apparently opposite effects of Notch on ferroptosis’ below.

Metabolic reprogramming serves as a central node for these interactions. MDM2/Notch interactions influence ferroptosis pathways,such as regulating the GPX4 and ACSL3 complex (Fu et al., 2024; Hua et al., 2024), by altering mitochondrial function (*e.g.*, reducing respiration) and lipid metabolism (Ma et al., 2024; Nakamura & Conrad, 2024). Taken together, the available data suggest that MDM2 and Notch can each affect ferroptosis through multiple, context-dependent routes, although formal demonstration of a unified co-regulatory circuit in a single system is still limited (Shi et al., 2024; Xia et al., 2024; Le et al., 2024).

The MDM2-p53 ferroptosis arm (Yin et al., 2024; Zhai et al., 2024) and Notch’s role in GPX4 regulation (Cai et al., 2024; Cao et al., 2024) have been explored largely in separate experimental systems, and an integrated picture of how the two pathways jointly modulate ferroptosis sensitivity *via* metabolic preferences in the TME remains to be established (Zhang et al., 2024; Zhao et al., 2024; Bhowmick et al., 2025). In the sections below, we describe the primary evidence without assuming integration, and we consider both iron/lipid metabolic rewiring and redox homeostasis within this framework (Gao et al., 2025; Guo et al., 2025).

### Ferroptosis in the tumor immune microenvironment

Metabolic reprogramming is a fundamental driver of tumor immune evasion. Ferroptosis has dual and opposing effects on anti-tumor immunity; the two arms below are each supported by independent studies rather than demonstrated within a single system, and we therefore present them as convergent observations rather than as one validated cascade. On one hand, ferroptotic cancer cells have been reported to release damage-associated molecular patterns (DAMPs), including HMGB1 and oxidized phospholipids, which in separate experimental systems can stimulate dendritic-cell maturation and CD8^+^ T-cell priming (Huang et al., 2025a; Kunishige et al., 2025); on the other, ferroptosis of tumor-infiltrating T cells, dendritic cells, and NK cells has been shown to impair their function and foster immune escape (Li et al., 2025; Wang et al., 2025c; Xu et al., 2025b; Chen et al., 2020). Whether these effects act sequentially within the same tumor has not been established. The net effect depends on the relative timing and magnitude of the two processes. MDM2 (through p53/SLC7A11) and Notch (through GPX4) each reshape ferroptosis susceptibility and can therefore indirectly influence this balance (Dai et al., 2020; Flerin et al., 2020).

Within the nutrient-scarce, hypoxic TME, metabolic stressors, low oxygen, and limiting amino acids, adenosine accumulation further skew this equilibrium. Notch-induced suppression of mitochondrial respiration (in DDLPS) and the general amino-acid scarcity of the TME can affect both tumor-cell ferroptosis sensitivity and immune-cell energy metabolism (Lee & Dixit, 2020; Wang et al., 2020; Bergers & Fendt, 2021). System x_c_^−^ inhibitors, for example, are reported to impair CD8^+^ T-cell effector function in addition to their anti-tumor effect (Jiang, Stockwell & Conrad, 2021; Ratnayake et al., 2021; Riefolo et al., 2021). Current literature addresses the general role of ferroptosis in the TME (Wang & Luo, 2021; Wei et al., 2021) and the MDM2/p53 axis (You et al., 2021; Zhang et al., 2021) largely in isolation; how these streams converge in human cancer remains to be tested directly (Lee & Dixit, 2020; Anonymous, 2022).

### Ferroptosis in the metastatic cascade

Ferroptosis is intimately linked to cancer metastasis. Reported mechanisms include ferroptosis effects on cancer-cell migration (Ala, 2022) and crosstalk with the epithelial–mesenchymal transition (EMT) programme *via* lipid metabolism and iron homeostasis reprogramming (Balihodzic et al., 2022; Chambers et al., 2022). As discussed in ‘Reported interactions between MDM2/Notch signaling and ferroptosis’, MDM2-mediated Numb degradation activates Notch, promoting stemness and potentially enhancing metastatic potential (Zhang et al., 2023b); ferroptosis resistance mechanisms (*e.g.*,  *via* MDM2/GPX4 Conway et al., 2022) protect metastatic cells from oxidative stress during dissemination (Covarrubias et al., 2022; Feng et al., 2022; Garg et al., 2022).

At the systems level, aberrant lipid metabolism coupled with EMT has been associated with ferroptosis resistance (Huppert et al., 2022; Jacobs et al., 2022), and MDM2 and Notch signaling each influence mitochondrial function and iron homeostasis in metastatic cells (Lee & Dixit, 2020; Kotschi et al., 2022; Li et al., 2022a). Preclinical work suggests that MDM2 inhibitors can restore ferroptosis sensitivity and may synergize with immunotherapy (Li et al., 2022b; Liao et al., 2022); however, clinical evidence for this combination is still limited (see ‘Therapeutic considerations and spatiotemporally-resolved strategies’ for detailed clinical-stage discussion). Most published work focuses either on ferroptosis in general (Lou et al., 2022; Luo et al., 2022) or on Notch signaling in specific cancers (Wang, Kavalali & Monteggia, 2022; Wang et al., 2022); studies that jointly perturb MDM2 and Notch in a metastatic-niche model are still scarce. Rather than presenting MDM2/Notch-ferroptosis crosstalk as a settled metastatic paradigm, we use the following sections to survey the primary evidence and to mark explicitly where extrapolation exceeds the data.

## Methods: literature search, inclusion criteria, and synthesis strategy

**Figure 1:**
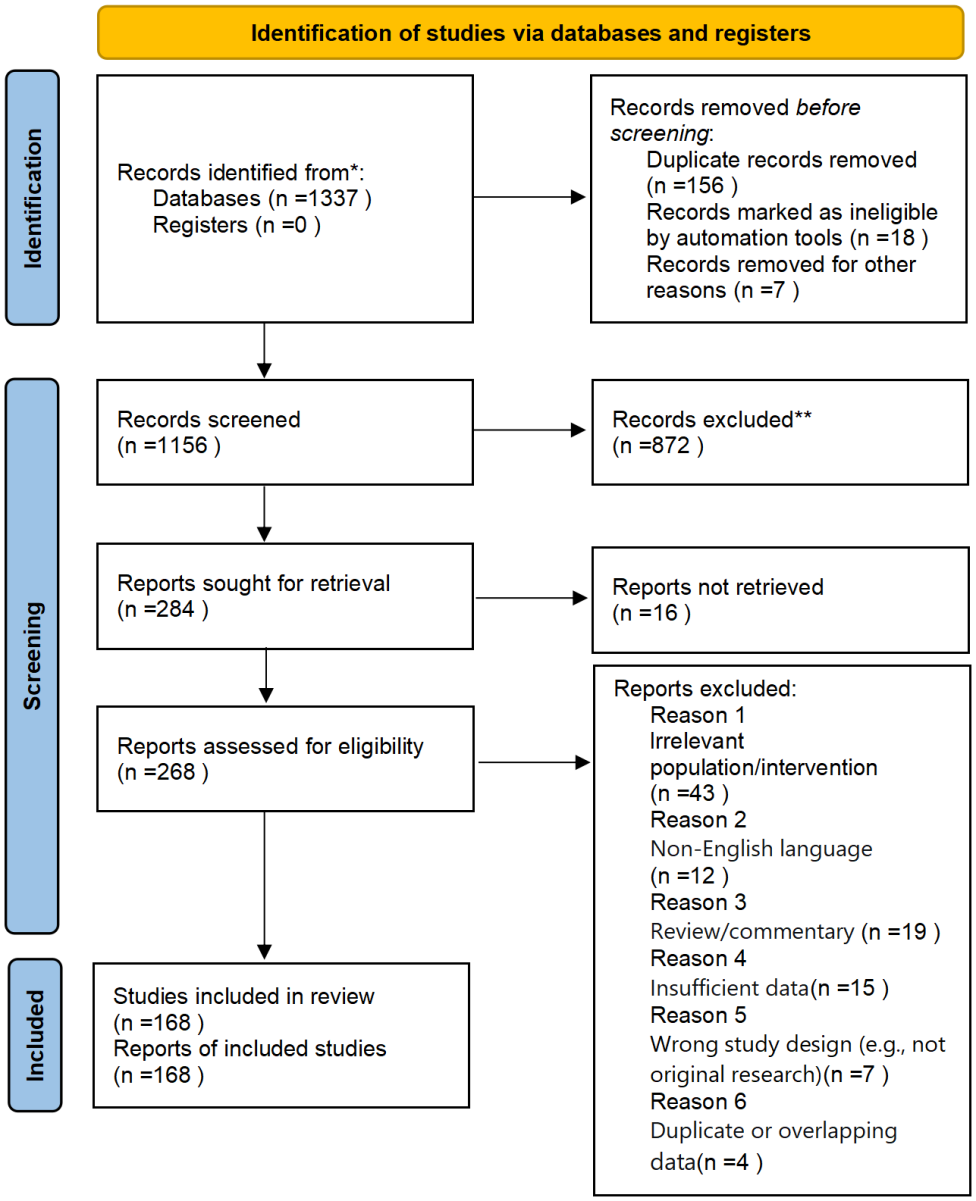
PRISMA 2020 flow diagram of study selection. After deduplication and screening of 1,337 records retrieved from three databases (2015–2024), 268 full-text reports were assessed for eligibility. Of these, 100 were excluded for the reasons specified in the diagram. A total of 168 studies were ultimately included in this review. Flowchart documents the screening process and does not represent a quantitative meta-analysis. *Consider, if feasible to do so, reporting the number of records identified from each database or register searched (rather than the total number across all databases/registers). **If automation tools were used, indicate how many records were excluded by a human and how many were excluded by automation tools.

This is a narrative review informed by a systematic search. We did not perform quantitative meta-analysis, and effect-size synthesis is therefore not reported. The PRISMA flow diagram (Fig. 1) is provided to document the search process transparently and does not imply quantitative pooling of effect estimates.

A comprehensive literature search was conducted across PubMed, Web of Science, and Scopus covering 1 January 2015 to 31 December 2024. The search strategy incorporated a combination of keywords and Medical Subject Headings (MeSH) terms related to *“MDM2,” “Notch signaling,” “ferroptosis,” “GPX4,” “SLC7A11,” “NUMB,” “AGPS,” “metabolic reprogramming,” “tumor immune evasion,”* and *“organotropic metastasis.”* A total of 1,337 records were identified through database searching. After removal of 156 duplicates and 25 records deemed ineligible by automation tools or for other reasons, 1,156 records were screened based on titles and abstracts. Following initial screening, 872 records were excluded as irrelevant. The full texts of the remaining 284 articles were sought for retrieval, of which 16 were not accessible. The remaining 268 articles underwent full-text eligibility assessment. Among these, 100 articles were excluded for the following reasons: irrelevant population or intervention (*n* = 43), non-English language (*n* = 12), review or commentary article without new synthesis (*n* = 19), insufficient data (*n* = 15), incorrect study design (*n* = 7), or duplicate/overlapping data (*n* = 4). Ultimately, 168 studies met the predefined inclusion criteria and were incorporated into this review.

### Inclusion criteria

Peer-reviewed, English-language articles published between 2015 and December 2024; original mechanistic or translational research, or authoritative review articles with original synthesis, that report on ≥1 of MDM2, Notch1/2/3/4, NUMB, AGPS, GPX4, SLC7A11, ACSL3/4, FSP1 or canonical ferroptosis readouts (lipid-ROS, 4-HNE, malondialdehyde, iron-dependent cell death rescuable by ferrostatin-1/liproxstatin-1); studies in cancer models, or studies in non-malignant models where the mechanistic logic plausibly extends to cancer (labeled and segregated in the synthesis).

### Exclusion criteria

Conference abstracts or preprints without full peer review; non-English articles; studies lacking primary mechanistic data beyond citation; commentaries or editorials without original synthesis; retracted articles.

### Synthesis strategy

Articles were grouped into four thematic matrices corresponding to ‘Molecular evidence for MDM2/Notch effects on ferroptosis–Open questions, experimental priorities, and limitations’ of this review: (i) molecular interactions; (ii) metabolic reprogramming; (iii) immune modulation; (iv) metastatic tropism. For each study, we extracted: experimental model system (species, tissue/cell line, cancer *vs* non-cancer, *in vivo vs in vitro*), direction of effect on the relevant readout, key molecular readouts used, and whether the finding was independently replicated in a second system. Apparent contradictions between studies were tabulated rather than resolved by selective citation. Findings from non-malignant systems are explicitly flagged (footnote † in Tables 1–2) and are described as plausibility-supporting rather than as direct evidence for cancer biology.

**Table 1:** Core molecular circuitry of the MDM2/Notch–ferroptosis axis. This table delineates key molecular regulators of the MDM2/Notch-ferroptosis network, with evidence stratified into two tiers: Mechanistically Coupled (causal link to ferroptosis validated in a single isogenic system) and Convergent Observation (correlative findings from independent studies, without unified mechanistic validation). For each entry, functional classification, direct targets, net effect on ferroptosis sensitivity, experimental models, and key references are provided.

**Molecule**	**Functional class**	**Direct target**	**Net effect on ferroptosis sensitivity**	**Evidence type**	**Representative experimental model(s)**	**Cancer specificity**	**Key reference(s)**
MDM2	E3 ubiquitin ligase	NUMB (ubiquitination & degradation)	Activates Notch signaling → inhibits ferroptosis	Mechanistically coupled	Colorectal cancer, cardiomyocytes, dedifferentiated liposarcoma (DDLPS)	Mixed (cancer + non-cancer^a^)	Zhang et al. (2023b), Jiang et al. (2023b) and Li et al. (2023b)
MDM2	E3 ubiquitin ligase	AGPS (ubiquitination & degradation)	Enhances ferroptosis susceptibility	Mechanistically Coupled	Prostate cancer cells	Cancer-specific	Zhang et al. (2024), Kim et al. (2023b) and Jiang et al. (2023c)
MDM2	E3 ubiquitin ligase	p53 (ubiquitination & degradation)	Blocks p53-mediated SLC7A11 downregulation → inhibits ferroptosis	Convergent Observation	Non-small cell lung cancer (NSCLC), acute kidney injury (AKI)^a^	Mixed (cancer + non-cancer^a^)	Lee & Roh (2023), Tien et al. (2023), Jin, Song & He (2023) and Mendiratta & Stites (2023)
Notch1	Transmembrane receptor/protein interactor	GPX4 (direct binding & stabilization)	Inhibits lipid peroxidation → master suppressor of ferroptosis	Mechanistically coupled	Myocardial ischemia/reperfusion (I/R) injury^a^, multiple tumor models	Mixed (cancer + non-cancer^a^)	Cai et al. (2024), Cao et al. (2024), Kumar & Stewart (2023) and Li et al. (2023a)
Notch1	Transcriptional regulator	PGC-1α (transcriptional repression)	Suppresses mitochondrial respiration → enhances ferroptosis susceptibility	Convergent observation	DDLPS	Cancer-specific	Zhang et al. (2023c) and Oria & Erler (2023)
p53	Transcription factor	SLC7A11 (transcriptional downregulation)	Impairs cystine uptake & GSH synthesis → sensitizes to ferroptosis	Convergent observation	NSCLC, colorectal cancer (CRC), AKI^a^	Mixed (cancer + non-cancer^a^)	Rodriguez, Schreiber & Conrad (2022), Zhang et al. (2022), Man et al. (2023) and Mendiratta & Stites (2023)
GPX4	Antioxidant enzyme	Lipid hydroperoxides (detoxification)	Constitutively suppresses ferroptosis (core negative regulator)	Convergent observation	Pan-cancer models, non-malignant cells	Universal	Cai et al. (2024), Cao et al. (2024), Man et al. (2023) and Miao et al. (2023)

**Notes.**

a denotes findings demonstrated only in non-malignant systems; cancer generalizability remains to be established (consistent with modification manuscript definition).

Abbreviations DDLPS dedifferentiated liposarcoma NSCLC non-small cell lung cancer CRC colorectal cancer I/R ischemia/reperfusion

**Table 2:** Metabolic hubs governing MDM2/Notch-dependent ferroptosis sensitivity. Key metabolic nodes (mitochondrial respiration, lipid metabolism, redox homeostasis, iron handling) that mediate MDM2/Notch-dependent ferroptosis modulation. For each node, regulatory effectors, direction of modulation, impact on ferroptosis sensitivity, tested models, and supporting references are listed.

**Metabolic node**	**Key gene/protein**	**Direction of regulation**	**Change in ferroptosis sensitivity**	**Exemplary cancer/tissue type(s)**	**Key reference(s)**
Mitochondrial respiration	PGC-1α	Notch-mediated transcriptional ↓	↑ Sensitivity	DDLPS	Zhang et al. (2023c) and Oria & Erler (2023)
Mitochondrial respiration	NDUFS1	↑ Mitochondrial translocation & restoration	↓ Sensitivity	Myocardial I/R injury^a^	Qi & Peng (2023)
Lipid metabolism & lipid-droplet biogenesis	ACSL3/ACSL4 axis	↑ PUFA esterification & lipid droplet synthesis	Context-dependent (stemness-associated ↓ sensitivity; proliferation-associated ↑ sensitivity)	Colorectal cancer, DDLPS	Lin et al. (2025a), Zhang et al. (2023b), Fu et al. (2024) and Hua et al. (2024)
Redox homeostasis & cystine/GSH metabolism	SLC7A11	p53-mediated transcriptional ↓	↑ Sensitivity	NSCLC, CRC	Rodriguez, Schreiber & Conrad (2022), Zhang et al. (2022), Thomas et al. (2023) and Wang et al. (2023a)
Redox homeostasis & antioxidant defense	GPX4	Notch1-mediated protein stabilization	↓ Sensitivity	Myocardial I/R injury^a^, multiple tumors	Cai et al. (2024), Cao et al. (2024), Kumar & Stewart (2023) and Li et al. (2023a)
Iron handling & iron homeostasis	TfR1/FTH1	↑ Labile iron uptake & storage	↑ Sensitivity	Breast cancer, NSCLC	Lin et al. (2023) and Li et al. (2024a)

**Notes.**

a denotes findings demonstrated only in non-malignant systems; cancer generalizability remains to be established.

Abbreviations PGC-1*α* peroxisome proliferator-activated receptor gamma coactivator 1-alpha NDUFS1 NADH: ubiquinone oxidoreductase core subunit S1 ACSL acyl-CoA synthetase long-chain family member SLC7A11 solute carrier family 7 member 11 GPX4 glutathione peroxidase 4 TfR1 transferrin receptor 1 FTH1 ferritin heavy chain 1

## Molecular evidence for MDM2/Notch effects on ferroptosis

MDM2 and Notch have each been reported to affect ferroptosis effectors through post-translational (ubiquitin-proteasome) and transcriptional mechanisms. In this section, we catalogue the primary observations by target and by model system, and indicate for each whether the interaction has been demonstrated within a single experimental system (mechanistically coupled) or inferred from parallel observations in separate systems (convergent).

For this review, an interaction is described as *mechanistically coupled* only when (i) perturbation of an MDM2-dependent event demonstrably alters a Notch-dependent event (or vice versa) within a single experimental system, and (ii) this coupling is accompanied by a measurable change in a canonical ferroptosis readout. Observations meeting only one of these criteria are described as *convergent* rather than coupled (Table 1).

### Core regulatory targets

Core molecular targets discussed here include MDM2, Notch signaling components (*e.g.*, Notch1), GPX4, and p53, which collectively modulate ferroptosis sensitivity through ubiquitination, protein stabilization, and transcriptional control.

**MDM2 as an E3 Ubiquitin Ligase Hub:** MDM2 has been reported to act on ferroptosis-related pathways *via* substrate ubiquitination and degradation. In cardiomyocytes and colorectal cancer models, MDM2 ubiquitinates and degrades NUMB, activating Notch signaling to inhibit ferroptosis and promote cancer stemness (Jiang et al., 2023b). In prostate cancer cells, MDM2 promotes ubiquitin-dependent degradation of AGPS (alkylglycerone phosphate synthase), a peroxisomal enzyme regulating ferroptosis sensitivity. Mechanistically, TrkA kinase-mediated phosphorylation of AGPS at Y451 facilitates its MDM2-dependent degradation, enhancing ferroptotic susceptibility (Jiang et al., 2023c). Conversely, in non-small cell lung cancer (NSCLC), miR-26a-1-3p targets MDM2 for silencing, stabilizing p53, downregulating SLC7A11, and triggering ferroptosis (Jin, Song & He, 2023). These findings establish MDM2 as a regulator of ferroptosis whose degradative activity is context-dependent and can in principle be therapeutically modulated (Kim et al., 2023a; Tang, Li & Liu, 2025).

**Notch Signaling as a Ferroptosis Suppressor:** Notch1 directly stabilizes GPX4 *via* interaction through its RAM domain, inhibiting ferroptosis (Kumar & Stewart, 2023). This mechanism is cardioprotective, as Notch1/Hes1 activation reduces lipid peroxidation and oxidative stress during myocardial ischemia/reperfusion (I/R) injury, notably *via* ZLY032 in a PPAR*δ*-dependent manner (Li et al., 2023a). In oncology, Notch activation frequently correlates with MDM2 upregulation, exemplified in dedifferentiated liposarcoma (DDLPS), which has been interpreted as a potential positive feedback loop in which MDM2-mediated NUMB degradation activates Notch, driving tumor aggressiveness (Jiang et al., 2023b; Li et al., 2023b), although formal demonstration of such a feedback loop in isogenic tumor models is still lacking. Notch signaling further inhibits ferroptosis through γ-secretase (Presenilin)-dependent cleavage of Notch-1 and downstream LRP8-mediated control of GPX4 expression (Lin et al., 2023).

**p53 and GPX4 as Regulatory Nodes:** The p53 tumor suppressor, regulated by the MDM2-p53 axis, is a key ferroptosis modulator. FTD/TPI treatment induces MDM2 ubiquitination and degradation, stabilizing p53 and promoting its nuclear accumulation. This downregulates SLC7A11 expression, culminating in ferroptosis (Man et al., 2023). A related MDM2–p53–LMNB1 axis has been described in acute kidney injury (AKI) † (Mendiratta & Stites, 2023); we note this as a non-cancer observation that supports plausibility but does not directly evidence cancer biology. GPX4, the central ferroptosis inhibitor, is regulated both directly and indirectly by the elements discussed above: Notch1 binding stabilizes GPX4 (Kumar & Stewart, 2023), while the MDM2-p53 axis induces the histone methyltransferase CARM1, which activates H3R26me2a methylation and transcriptionally upregulates GPX4 (Cheng et al., 2023; Miao et al., 2023; Hirata et al., 2024).

**Figure 2:**
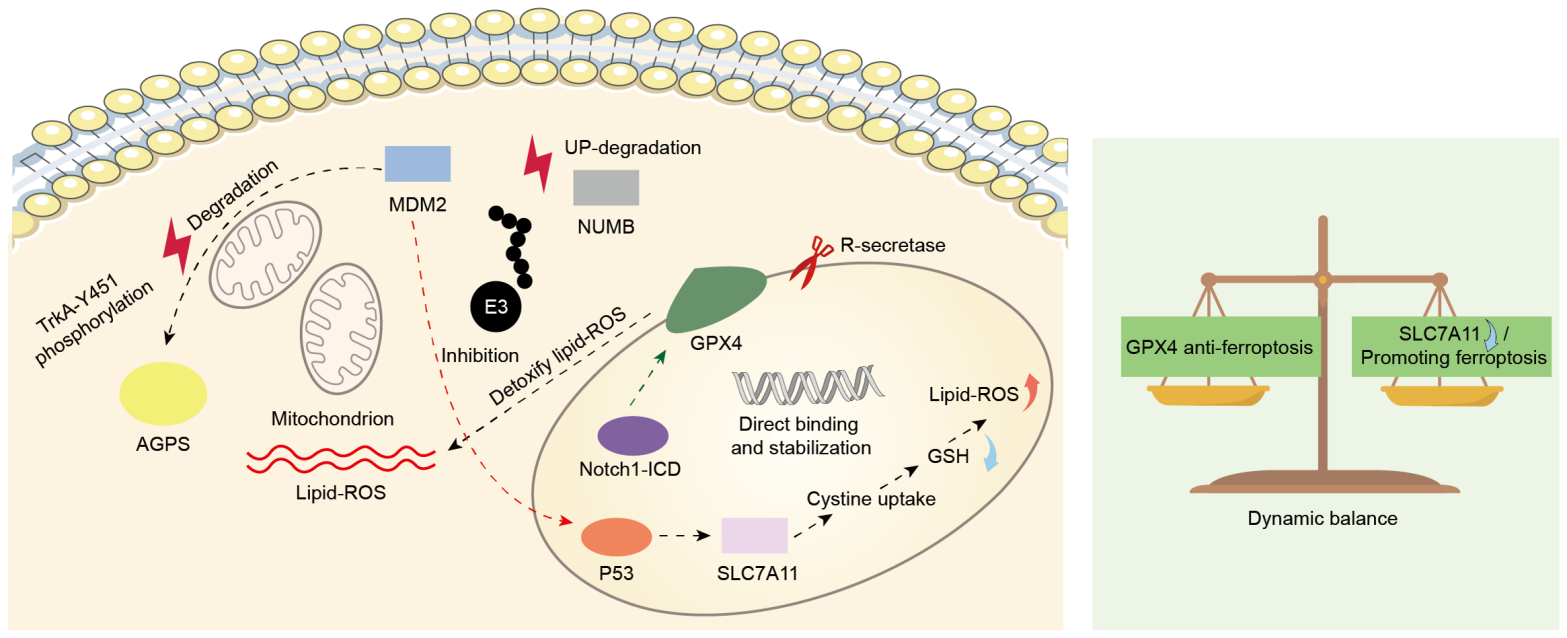
MDM2–Notch crosstalk in ferroptosis regulation. MDM2 promotes ubiquitin-dependent degradation of NUMB, relieving Notch inhibition; the released Notch1 intracellular domain (Notch1-ICD) directly binds and stabilizes GPX4 to suppress lipid-ROS. MDM2 also targets AGPS for degradation and, through p53, transcriptionally represses SLC7A11, thereby limiting cystine uptake and GSH synthesis to impair GPX4 and promote ferroptosis. Solid arrows indicate mechanistically coupled interactions demonstrated within a single experimental system; dashed arrows denote convergent findings from independent studies. The net ferroptotic outcome reflects the dynamic balance between these opposing effects.

In summary, MDM2, Notch1, p53, and GPX4 constitute interacting regulatory nodes whose effects on ferroptosis sensitivity have been documented individually in diverse systems. A unified circuit in which all four are simultaneously perturbed in a single tumor model has not yet been reported; Fig. 2 therefore distinguishes solid arrows (mechanistically coupled, demonstrated within a single system) from dashed arrows (convergent, inferred from independent studies).

### Metabolic reprogramming nodes

The above interactions profoundly intersect with metabolic processes centered on mitochondrial function, redox homeostasis, and lipid metabolism, which in turn feed back on ferroptosis sensitivity.

**Mitochondrial metabolism.** Mitochondria exert multifaceted control over ferroptosis through oxidative phosphorylation and respiration. In DDLPS, Notch activation correlates with suppressed mitochondrial respiration, heightened oxidative stress, and increased ferroptosis susceptibility (Oria & Erler, 2023). Conversely, Lutein@DTPP counteracts cardiomyocyte ferroptosis † by facilitating NDUFS1 mitochondrial translocation (Qi & Peng, 2023); we flag this as a non-cancer finding that supports the general principle but has not been tested in a tumor model. Ferroptosis in vascular smooth muscle cells is similarly linked to electron-transport-chain disruption (Qin et al., 2023). Across systems, mitochondrial reprogramming (of amino-acid, lipid, and glucose metabolism) can drive lipid peroxidation accumulation (Ji et al., 2023; Ren et al., 2023).

**Redox homeostasis and lipid peroxidation.** Notch signaling has been reported to bolster antioxidant defenses, primarily by stabilizing GPX4 to detoxify lipid hydroperoxyl radicals, thereby inhibiting lipid peroxidation and ferroptosis (Kumar & Stewart, 2023; Lin et al., 2023). This pathway is critically protective in myocardial I/R injury, where Notch1/Hes1 activation mitigates lipid peroxidation and oxidative damage (Li et al., 2023a). Concurrently, the MDM2-regulated p53 axis disrupts redox balance: p53 nuclear accumulation downregulates SLC7A11, impairing cystine uptake and glutathione (GSH) synthesis, elevating oxidative stress, and precipitating ferroptosis (Thomas et al., 2023; Wang et al., 2023a). In cancer, non-canonical NRF2/NOTCH interactions have been described that reshape amino-acid and lipid metabolism (Wang et al., 2023b). In non-malignant intestinal stem cells †, iron overload induces oxidative stress and ferroptosis while inhibiting Notch signaling (Wu et al., 2023; Wan et al., 2025), again a non-cancer system that we cite for mechanistic plausibility (Table 2).

**Integration of metabolic networks.** Collectively, the data above suggest that MDM2 and Notch modulate metabolism by influencing selenium utilization (GPX4), iron ion dynamics, and ROS generation (Li et al., 2024a). Dysregulation of ferroptosis-related proteins (MDM2, SLC7A11, GPX4) in AML12 hepatocytes † has been proposed as an indicator of redox imbalance (Xiao et al., 2023), though direct cancer validation is still pending.

### Reconciling apparently opposite effects of Notch on ferroptosis

Notch has been reported both to inhibit ferroptosis (*via* GPX4 stabilization, ‘Core regulatory targets’) and to increase ferroptosis susceptibility (*via* mitochondrial-respiration suppression in DDLPS). This apparent paradox is not resolved by selecting one set of studies over another. We instead propose a working model in which four contextual variables determine the net direction of Notch’s effect:

**1. Cell lineage and baseline GPX4 dependence.** In differentiated cells that depend heavily on GPX4 to maintain membrane integrity under oxidative stress (cardiomyocytes under I/R, Li et al., 2023a), Notch1/Hes1-driven GPX4 stabilization is net-protective. In rapidly proliferating tumor cells with high oxidative-phosphorylation demand (DDLPS, Oria & Erler, 2023), Notch-mediated PGC-1α repression reduces mitochondrial biogenesis and NADPH-regenerating capacity, and the loss of reducing equivalents outweighs the GPX4 stabilizing effect.

**2. Downstream transcriptional partners.** Notch’s antioxidant output depends on NICD/MAML/RBP-J*κ* access to Hes-family promoters; its pro-ferroptotic output depends on repression of PGC-1α and on NRF2 crosstalk. In KEAP1-mutant lung cancers with constitutively active NRF2 (Sparaneo et al., 2025), antioxidant defense dominates; where NRF2 activity is low, Notch-driven metabolic reprogramming predominates and ferroptosis sensitivity rises.

**3. Oxygen tension.** In hypoxia, mitochondrial respiration is already suppressed, and the “respiration-dampening” arm of Notch has little additional effect; GPX4 stabilization dominates. In normoxia, Notch-induced respiration suppression has full effect and can overwhelm GPX4 stabilization. This is consistent with the observation that ferrostatin-1 rescues Notch-active DDLPS cells more efficiently in normoxia than in hypoxia (Oria & Erler, 2023).

**4. Ligand/receptor stoichiometry.** DLL4-driven lateral inhibition produces short, sharp NICD pulses that favor stemness and GPX4 stabilization; Jagged-driven lateral induction yields sustained low-amplitude signaling that biases toward metabolic reprogramming.

We note that this model is a working hypothesis derived from cross-referencing independent studies rather than a validated framework. Experiments that would test it include isogenic DDLPS and cardiomyocyte lines with matched GPX4 and PGC-1α readouts under paired normoxic/hypoxic conditions, and across DLL4- *versus* Jagged-dominated ligand stimulation.

## Intersection with immune evasion and organ-selective metastasis

Tumor metabolic reprogramming is intricately linked to immune responses and metastatic dissemination. Three specific molecular bridges connect the processes discussed in ‘Core regulatory targets–Metabolic reprogramming nodes’ to immune evasion and metastasis: (i) lipid-peroxidation flux links GPX4 status to DAMP release and thence to innate-immune sensing; (ii) cystine/GSH availability *via* SLC7A11 links amino-acid metabolism to CD8^+^ T-cell fitness and to the oxidative-stress tolerance required for metastatic survival; (iii) iron handling *via* TfR1 and FTH1 links iron metabolism to pre-metastatic niche formation, particularly in GPX3^+^ alveolar type II cells. Beyond fueling energy and biosynthetic demands, metabolic alterations actively reshape the TME (Yan et al., 2023; Zhai et al., 2023; Zhang et al., 2023a). Immunometabolic checkpoints critically govern the fate of glucose, lipids, and proteins, thereby influencing both immune cell efficacy and metastatic potential (Zheng et al., 2023). The following subsections detail the roles of immune evasion and organ-specific metastasis within this framework.

### Metabolism-linked immune evasion mechanisms

Immune evasion can be influenced by metabolic reprogramming, a relationship critically important during metastatic progression. For each mechanism below, we indicate the evidence tier with respect to MDM2/Notch–ferroptosis regulation: A = directly demonstrated link; B = inferred from shared downstream effectors; C = currently speculative.

Adenosine signaling (tier B). CD73 (ecto-5′-nucleotidase)-generated adenosine exerts potent immunosuppressive effects and impairs natural killer (NK) cell function, conferring an immune-escape advantage to tumor cells (Bai et al., 2024). CD73 is a p53 target gene, and p53 is MDM2-regulated; however, no study to our knowledge has demonstrated that MDM2 inhibition modulates CD73 in a ferroptosis-dependent manner, so we describe this link as inferred rather than direct.

Immune checkpoint upregulation. Chronic activation of the cGAS-STING pathway has been associated with elevated immune checkpoint expression (*e.g.*, PD-L1), suppressing T cell activity and promoting both immune evasion and metastatic spread (Berndt et al., 2024). The individual steps, GPX4 loss and lipid peroxidation, ferroptotic DAMP/dsDNA release, cGAS–STING engagement, and PD-L1 upregulation, are each supported in the literature but have not all been demonstrated as a single continuous pathway in one model. The link we classify as tier A (directly demonstrated) is specifically the observation of Wei et al. (2024), in which a ferroptosis-boosting exosome engaged cGAS–STING and elicited PD-L1 upregulation within one experimental system; the upstream connection from GPX4 status to DAMP release is assembled from independent studies and is therefore tier B. In hepatocellular carcinoma (HCC), TKT protein activation *via* the ROS-mTOR axis has been reported to increase PD-L1 and VRK2 expression, driving immune escape and metastasis (Chen et al., 2024; Li et al., 2024b) (Table 3).

Extracellular vesicle (EV) mediation (tier C). Tumor-derived EVs modulate immunosuppressive pathways (Jiang et al., 2023a; He et al., 2024). Ferroptotic cells release distinct EV cargoes (Kuang, Wu & Li, 2025), but these have not been mechanistically tied back to MDM2 or Notch, so we retain this mechanism as speculative.

Hypoxia and HIF-1 signaling (contextual modifier). Within the hypoxic TME, HIF-1 orchestrates processes critical for immune evasion (Jang et al., 2024). We describe hypoxia as a converging modifier rather than a downstream effect of MDM2/Notch signaling, consistent with the oxygen-tension argument in ‘Reconciling apparently opposite effects of Notch on ferroptosis’.

### Organ-selective metastasis

Organ-specific metastasis, the preferential colonization of tumor cells in distinct organs (*e.g.*, lymph nodes, lungs, liver), is finely regulated by reciprocal metabolism–immune interactions. Metabolic reprogramming is integral to each step of the metastatic cascade, while the unique metabolic and immune landscape of target organ microenvironments dictates metastatic tropism.

**Table 3:** Immune evasion programs linked to the MDM2/notch–ferroptosis axis. Metabolism-driven immune evasion mechanisms downstream of the MDM2/Notch-ferroptosis axis, with evidence stratified into 3 tiers: Tier A (direct causal linkage validated), Tier B (inferred from shared downstream effectors), Tier C (speculative, no definitive experimental support). For each mechanism, metabolic drivers, impaired immune populations, effector pathways, and clinically targetable agents are provided.

**Evasion mechanism**	**Metabolic driver**	**Impaired immune population**	**Effector molecule/pathway**	**Evidence tier**	**Clinically targetable marker/agent**
Adenosinergic immunosuppression	CD73 overexpression (p53 target gene)	NK cells, CD8^+^ T cells	CD73/A2A receptor signaling	Tier B (inferred from shared effectors)	CD73 monoclonal antibody
Immune checkpoint upregulation	Chronic cGAS–STING activation by ferroptotic DAMPs	CD8^+^ T cells	PD-1/PD-L1 axis	Tier A (directly demonstrated)	PD-1/PD-L1 inhibitors
T cell functional impairment & ferroptosis	System Xc^−^ inhibition & cystine deprivation	CD8^+^ T cells, dendritic cells	GPX4 depletion, lipid ROS accumulation	Tier B (inferred from shared effectors)	Ferrostatin-1, cell-specific ferroptosis prodrugs
Intrahepatic immune suppression	FBXL6–TKT–ROS–mTOR metabolic loop	Intrahepatic CD8^+^ T cells	PD-L1 upregulation cascade	Tier B (inferred from shared effectors)	TKT inhibitors + PD-1 blockade
Extracellular vesicle (EV)-mediated immunosuppression	Ferroptotic tumor cell-derived EV cargo transfer	Tumor-infiltrating immune cells	Unspecified immunosuppressive cargo	Tier C (speculative)	No validated clinical agent

**Notes.**

Evidence Tier definition (consistent with modification manuscript): A = directly demonstrated link to MDM2/Notch-ferroptosis axis; B = inferred from shared downstream effectors; C = currently speculative.

Abbreviations NK natural killer DAMPs damage-associated molecular patterns cGAS-STING cyclic GMP-AMP synthase–stimulator of interferon genes PD-1 programmed cell death protein 1 PD-L1 programmed death-ligand 1

**Lymph node metastasis.** In cervical cancer, downregulation of the lncRNA LNMAS promotes EMT and lymph node metastasis *via* immune-evasion mechanisms (Li et al., 2024c). Lymph nodes foster immune tolerance through regulatory T cell (Treg) accumulation (Liu et al., 2024a).

**Lung metastasis.** Breast cancer lung colonization relies on the metabolic activity of resident stromal cells, which establish an immunosuppressive niche (Liu et al., 2024b; Liu et al., 2024c). Obesity-associated systemic metabolic alterations further promote lung metastasis (Luo et al., 2024).

**Liver metastasis.** Liver metastasis in HCC is critically dependent on immune evasion; the FBXL6-TKT axis promotes immune suppression within the liver microenvironment (Chen et al., 2024; Meng, Wei & Shan, 2024). HLA loss of heterozygosity in lung adenocarcinoma metastasis (Chen et al., 2023b; Piening et al., 2024) illustrates microenvironment-driven selection.

**Contextual modifiers.** Obesity-associated metabolic rewiring (Su et al., 2024), TSPAN4-positive fibroblasts in pancreatic cancer (Tang, Kroemer & Kang, 2024), and 9p21.3 IFN-cluster deletion (Wang et al., 2024a) have each been linked to immune evasion and metastasis but are not known to be downstream of MDM2 or Notch (Table 4).

**Table 4:** Organ-specific metastasis: metabolic-immune signatures and ferroptosis linkage. Organ-specific metabolic adaptations, immune microenvironment features, and ferroptosis regulatory programs enabling metastatic colonization of lymph nodes, lung, and liver. For each site, master regulatory axes, functional linkage to the MDM2/Notch-ferroptosis network, and candidate intervention strategies are detailed.

**Metastatic organ**	**Metabolic adaptation**	**Immune microenvironment feature**	**Master regulatory axis**	**Ferroptosis linkage**	**Exemplary intervention strategy**
Lymph nodes	EMT + *de novo* lipogenesis + lipid droplet accumulation	Regulatory T cell (Treg) accumulation, immune tolerance induction	LNMAS downregulation → EMT activation; MDM2-NUMB-Notch axis	Notch activation drives ferroptosis resistance & stemness, enabling lymph node colonization	Targeting lncRNA LNMAS; MDM2 inhibitors
Lung	Alveolar lipid droplet utilization, kynurenine-mediated metabolic rewiring	Alveolar macrophage M2 polarization, GPX3^+^ alveolar type II (AT2) cell pre-metastatic niche formation	GPX3^+^ AT2 stromal cell axis; Tryptophan 2,3-dioxygenase metabolic loop	Metastatic cells acquire GPX4-mediated ferroptosis resistance to survive oxidative stress in lung niche	Inhibiting alveolar lipid metabolism; GPX4 targeted therapy
Liver	Pentose phosphate pathway activation via FBXL6-TKT axis	Kupffer cell depletion, impaired antigen presentation	FBXL6–TKT–ROS–mTOR–PD-L1 cascade	ROS accumulation drives ferroptosis sensitivity modulation & immune evasion	TKT inhibitor + PD-1 blockade; MDM2-p53 axis targeted therapy

**Notes.**

Abbreviations EMT epithelial–mesenchymal transition AT2 alveolar type II lncRNA long non-coding RNA

**Figure 3:**
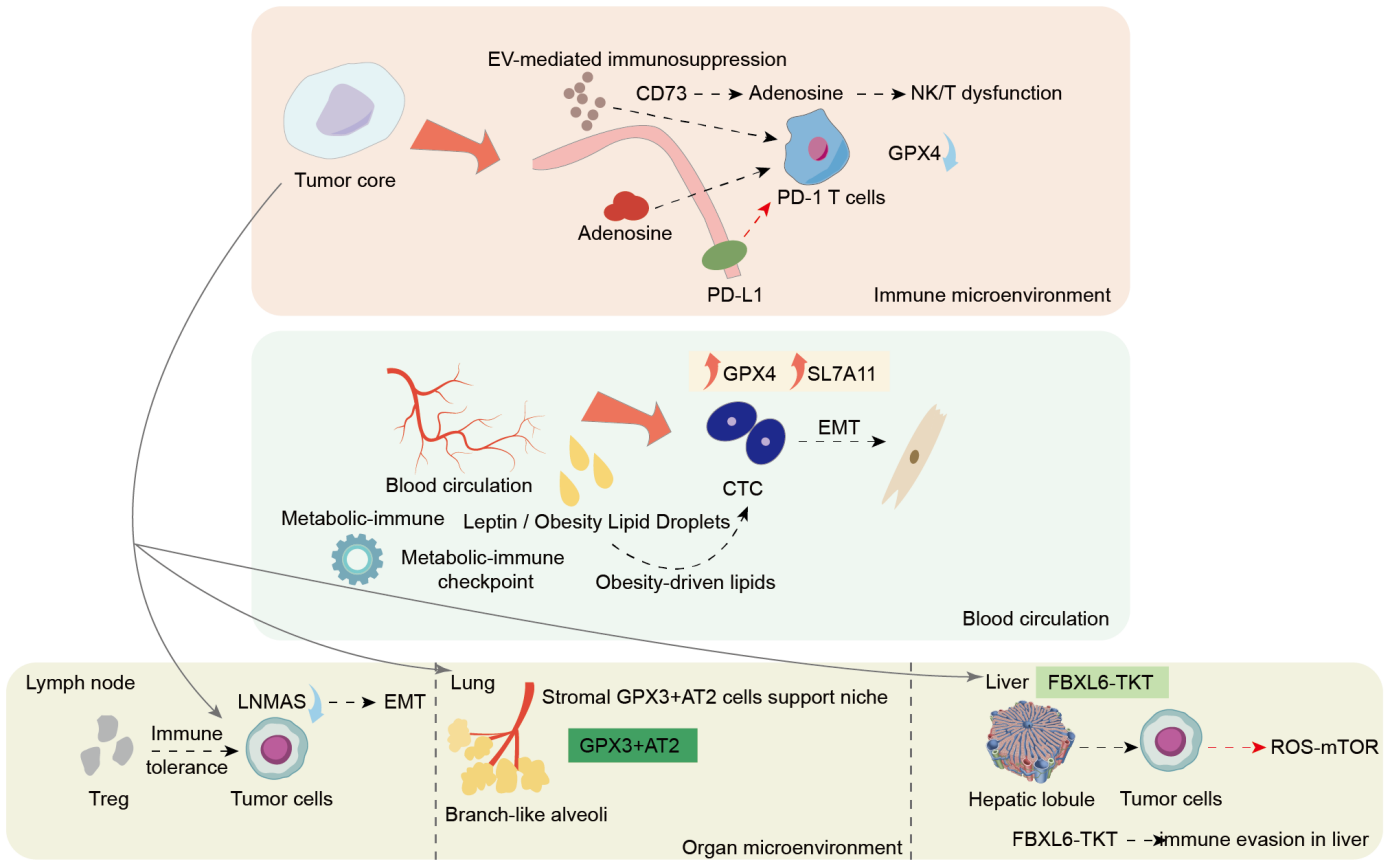
Ferroptosis at the metabolic–immune–metastasis axis. Ferroptosis-related metabolic changes link immune evasion to organotropic metastasis. In the tumor core, GPX4-stabilized cells release EVs; CD73-generated adenosine and PD-L1 upregulation suppress T- and NK-cell function. Obesity-driven lipids and leptin alter immune-cell metabolism. Lymph node metastasis involves LNMAS downregulation and Treg-mediated immune tolerance. GPX3+ AT2 cells support the lung pre-metastatic niche. In the liver, the FBXL6–TKT axis activates ROS–mTOR signaling to drive immune escape. SLC7A11, GPX4, and EMT connect ferroptosis resistance to metastatic progression. Dotted arrows denote routes for which a direct mechanistic link to MDM2 or Notch remains to be established.

These organ-specific patterns underscore metabolic reprogramming as a contributor to metastasis, and spatial transcriptomic and metabolomic studies will be needed to map organ-specific metabolic-immune interactions at single-cell resolution (Wang et al., 2024b; Wang et al., 2024c) (Fig. 3).

## Therapeutic considerations and spatiotemporally-resolved strategies

Targeted therapies have expanded oncology by exploiting molecular or cellular vulnerabilities with unprecedented precision. Nevertheless, their clinical benefit is frequently curtailed by paradoxical effects, pathway re-activation, drug-resistance rebound, and systemic toxicity. Spatiotemporally resolved interventions, which dictate where and when a drug exerts its action, offer a conceptual and technological framework to mitigate these paradoxes. This section critically synthesises recent literature on the mechanistic origins of these challenges and the emerging strategies that exploit spatiotemporal control.

### Clinical-stage status and mechanistic barriers

It is important to state at the outset that no MDM2 inhibitor has yet been approved for oncology, and no bona-fide ferroptosis inducer has entered registration-directed clinical trials. Idasanutlin (RG7388) did not meet primary endpoints in the MIRROS phase III AML trial; milademetan (DS-3032b) did not reach primary endpoints in the MANTRA liposarcoma trial; APG-115 and KRT-232 remain in earlier-phase development. On-target MDM2 blockade is associated with dose-limiting thrombocytopenia, neutropenia, and GI toxicity. Ferroptosis-inducing activity has been reported for approved agents such as sulfasalazine (SLC7A11 inhibitor) and artemisinin derivatives, but these were not developed for that indication and repurposing evidence is mixed. Importantly, “ferroptosis sensitivity” as a patient-selection parameter is measured only in preclinical models at present and has no clinically validated assay.

**Paradoxical activation.** In the RAS/RAF axis, ATP-competitive RAF inhibitors such as vemurafenib can trigger transactivation of wild-type RAF dimers, culminating in MEK/ERK hyperactivation (Chai et al., 2023; Wang et al., 2024d). We include this example only as a broader conceptual precedent; the specific phenomenon of Notch rebound after MDM2 inhibition is discussed below. Analogously, checkpoint inhibitors combined with cytotoxic chemotherapy may blunt anti-tumour immunity (Wei et al., 2024).

**Drug resistance and rebound.** Combination regimens that pair immune checkpoint blockade with cytotoxics escalate grade 3–4 toxicities without proportional efficacy gains (Hong et al., 2024; Wu et al., 2024). Resistance can emerge through tumor-microenvironment co-evolution (Yan et al., 2024; Sun et al., 2025).

**Systemic toxicity paradox.** Conventional systemic administration exposes off-target tissues, generating burdensome toxicities. Adjuvant endocrine therapy (AET) exemplifies this dilemma (Yang et al., 2024). Whole-body metabolic interventions intended to starve tumors can inadvertently fuel metastatic dissemination (Zhou & Yu, 2024) (Table 5).

**Table 5:** Therapeutic paradoxes and spatiotemporally resolved solutions for MDM2/Notch-Ferroptosis targeting. Key paradoxical effects limiting clinical translation of MDM2/Notch-ferroptosis-targeted therapies, including their molecular basis, and emerging spatiotemporally controlled strategies to mitigate these limitations. Preclinical proof-of-concept models for each strategy are also listed.

**Treatment class**	**Paradoxical effect**	**Molecular basis**	**Targeted problem in MDM2/Notch-Ferroptosis Axis**	**Spatiotemporal intervention technology**	**Pre-clinical proof-of-concept model**
MDM2 inhibitors	Notch rebound & therapy resistance	Systemic MDM2 inhibition relieves NUMB degradation in normal stem-cell niches, driving off-target Notch activation	Notch rebound after systemic MDM2 inhibition	NIR-activatable PROTAC-mediated focal MDM2 degradation	Orthotopic triple-negative breast cancer (TNBC) mouse model
Systemic ferroptosis inducers (GPX4 inhibitors)	Collateral killing of anti-tumor immune cells	Pan-tissue GPX4 suppression impairs CD8^+^ T cell, dendritic cell & NK cell fitness	Off-target ferroptosis in tumor-infiltrating immune cells	ROS-cleavable hydrogel for tumor-localized drug release; cell-specific ferroptosis prodrugs	CT26 colorectal cancer liver-metastasis model
Ferroptosis induction + immune checkpoint blockade (ICB)	Compensatory PD-L1 upregulation & aggravated immune tolerance	Ferroptotic DAMPs chronically activate cGAS–STING, driving PD-L1 upregulation on surviving tumor cells	Compensatory PD-L1 upregulation after ferroptosis induction	Ultrasound-responsive microbubble platform for temporally synchronized anti-PD-L1 focal delivery	4T1 breast cancer lung-metastasis model
Systemic metabolic therapy	Paradoxical acceleration of distant metastasis	Whole-body lipid & amino acid metabolic rewiring enhances metastatic niche fitness	Systemic metabolic intervention fuels organ-specific metastasis	NRP-1/transferrin receptor-targeted organ-restricted nanocarriers	Obesity-associated breast cancer metastasis model

**Notes.**

Abbreviations MDM2 mouse double minute 2 homolog ICB immune checkpoint blockade PROTAC proteolysis-targeting chimera NIR near-infrared TNBC triple-negative breast cancer

### Spatiotemporally-resolved strategies mapped to axis-specific problems

**Problem 1: Notch rebound after MDM2 inhibition.** Systemic MDM2 inhibitors relieve NUMB degradation but also lift MDM2 repression of Notch target genes in non-tumor stem-cell niches. NIR-activatable MDM2-PROTAC constructs (Wang et al., 2023a) confine MDM2 degradation to illuminated tumor foci and minimize Notch rebound in GI/hematopoietic tissue.

**Problem 2: off-target ferroptosis in immune cells.** GPX4 inhibitors also deplete CD8^+^ T cells and dendritic cells (Anonymous, 2022; Liu et al., 2024b). Cell-specific ferroptosis prodrugs (Wang et al., 2025c) and ROS-cleavable hydrogels (diselenide linkers) (Hu et al., 2025) release GPX4 inhibitors in tumor beds while sparing systemic immune-cell GPX4. Injectable composite hydrogels loaded with chemo-immunomodulatory cargoes (Chen et al., 2023a; Chen et al., 2025) have shown feasibility in resected triple-negative breast cancer beds.

**Problem 3: compensatory PD-L1 upregulation after ferroptosis induction.** Ferroptotic DAMPs activate cGAS–STING and can elevate PD-L1 on surviving cells. Ultrasound-responsive microbubbles (Jiang, Liu & Sun, 2025) and ultrasound-programmable delivery platforms (Dai et al., 2023; Wang et al., 2025b) allow focal deposition of anti-PD-L1 cargo to be temporally synchronized with the ferroptosis induction pulse.

**Problem 4: systemic metabolic rewiring fuelling metastasis.** Because whole-body metabolic therapy can paradoxically accelerate metastasis (Zhou & Yu, 2024), organ-restricted nanocarriers, for example, NRP-1-targeted FPPT@Axi (Huang et al., 2025b) and transferrin-receptor-targeted constructs (Yan et al., 2024), allow MDM2 inhibitors to reach metastatic lesions while sparing metabolically active normal organs.

**Photocontrolled systems and photodynamic therapy.** Near-infrared (NIR) activatable constructs provide sub-millimetre spatial resolution. The autonomous singlet-oxygen nanogenerator (aSOND) (Zhou et al., 2024a) confines cytotoxic ROS to illuminated foci. Orthogonal upconversion nanoparticles (OUNCs) conjugated to photosensitisers enable spatiotemporally precise gene silencing (Zhou et al., 2024b). Photocaged PROTACs that degrade BRD4 only upon light exposure (Zhou et al., 2024c; Zhou & Lin, 2024; Lin et al., 2025b) illustrate the concept extensible to MDM2.

Practical barriers worth flagging: PROTAC oral bioavailability and half-life remain limiting; hydrogel manufacturing and regulatory pathways for implantable depot systems are immature; tumor heterogeneity and the absence of a clinical ferroptosis-sensitivity assay mean that biomarker-guided patient selection is still aspirational.

Integration and future directions. A pragmatic framework for spatiotemporally-resolved therapy of the MDM2/Notch–ferroptosis interaction would combine three modules: targeted delivery, microenvironment modulation, and cell-interaction steering (Lv, Wu & Chen, 2025). Multi-omic atlases of primary and metastatic lesions will be needed to calibrate these modules (Sparaneo et al., 2025; Oh et al., 2025; Peng-Winkler & Fendt, 2025; Qian et al., 2025; Ran et al., 2025; Sears & Carter, 2025; Shi et al., 2025). Machine-intelligence-guided adaptive dosing (Thakor, 2025) is plausible but has not yet been validated prospectively in oncology.

## Open questions, experimental priorities, and limitations

### Open biological questions

Several basic questions remain open. First, no published study has simultaneously perturbed MDM2, Notch, and a ferroptosis effector (GPX4 or SLC7A11) within one isogenic tumor model. Studies that would address this gap could compare MDM2-null, Notch1-null, and double-knockout isogenic pairs across ≥5 cancer types (breast, lung, colorectal, hepatocellular, pancreatic). Second, the context-dependent effects of Notch on ferroptosis (‘Reconciling apparently opposite effects of Notch on ferroptosis’) have not been tested systematically under matched oxygen tensions or with paired DLL4/Jagged stimulation; such experiments would test the unifying model proposed in this review. Third, the dual role of ferroptosis in immunity, immunogenic tumor-cell ferroptosis *versus* immune-cell-impairing ferroptosis, remains a quantitative problem rather than a qualitative one; the relative timing and magnitude of the two processes, not their mere co-occurrence, dictate the clinical outcome.

### Translational considerations and biomarker gaps

Clinical translation of agents targeting this network requires validated, analytically robust biomarkers that are not yet available. Composite signatures such as low GPX4 plus high 4-HNE plus high CD8^+^ T-cell density have been proposed (Wang et al., 2025b; Wang et al., 2025a; Xie et al., 2025a; Xie et al., 2025b), and *in silico* scores such as the Fersig score exist, but none have been prospectively validated in a registrational trial. Multiplexed imaging mass cytometry (Xu et al., 2025a; Yan et al., 2025) using lipid-free and Fe-chelated buffers will be needed to avoid *ex vivo* oxidation artefacts that presently compromise ferroptosis biomarker measurement. Photothermal therapy combined with GPX4 inhibitors has shown feasibility for immunogenic cell death with CD8^+^ T-cell preservation in preclinical metastasis models (Huang et al., 2023; Yue et al., 2025), but dose-titration approaches that protect immune-cell fitness (Zhang et al., 2025b) must be refined before clinical application.

### The ferroptosis–immunity paradox

Ferroptosis-induced DAMPs activate cGAS–STING and prime T cells (Yang et al., 2025), but ferroptotic death of dendritic and CD8^+^ T cells impairs immune memory (Yi et al., 2025). Resolving this tension will likely require combination regimens that decouple the two outcomes: for example, IDO blockade or calibrated cGAS–STING agonism (Zhang et al., 2025a) to protect immune effectors, followed by sequenced immune-checkpoint blockade (CD73/PD-L1) to prevent relapse. Real-time imaging of labile iron and lipid-ROS kinetics would be needed to guide such dosing and to convert acute inflammation into durable anti-tumor memory without immune exhaustion (Jing et al., 2025).

### Limitations of this review

Several limitations must be acknowledged. The majority of existing evidence is derived from preclinical models, cell lines, and animal studies, which may not fully capture the complexity of human tumors. Clinical validation of MDM2/Notch–ferroptosis interactions in organ-specific metastasis and immune evasion remains scarce. The dual role of ferroptosis (both immune-promoting and immunosuppressive) complicates therapeutic targeting, and the spatiotemporal dynamics of ferroptosis induction *in vivo* are incompletely elucidated. Emerging tools, single-cell multi-omics, and spatially resolved imaging hold promise, but the instability of labile metabolites such as lipid radicals and Fe^2+^ during sample processing compromises data integrity. Finally, the translational potential of NIR-activated PROTACs and ROS-responsive hydrogels requires refinement of scalability, biocompatibility, and release-kinetics control before clinical deployment (Wang et al., 2021; Kim et al., 2023b; Xu et al., 2025b; Wang et al., 2023a; Tang, Kroemer & Kang, 2024; Lee et al., 2024).

## Summary

This review has surveyed the primary evidence linking MDM2 and Notch signaling to ferroptosis regulation in cancer, and has examined how the metabolic consequences of these interactions intersect with tumor immune evasion and organ-selective metastasis. At the molecular level, MDM2-dependent ubiquitination of NUMB and AGPS, Notch1-GPX4 protein interaction, the MDM2–p53–SLC7A11 axis, and NRF2/NOTCH crosstalk have each been documented individually; a unified circuit demonstrated in a single tumor model is not yet available, and Notch’s effect on ferroptosis is strongly context-dependent (‘Reconciling apparently opposite effects of Notch on ferroptosis’). Within the immune microenvironment, metabolic stress imposed by this network, hypoxia, amino-acid scarcity, and adenosine accumulation can weaken T-cell effector programmes, although not all reported immune-evasion mechanisms have been mechanistically tied to MDM2 or Notch. At distant sites, metabolic adaptation dovetails with organ-specific niches (lung stromal cells, hepatic FBXL6–TKT axis) to enable selective colonization. Therapeutically, MDM2 inhibitors and ferroptosis inducers have promise but have not yet produced positive registrational oncology trials; systemic toxicity and spatiotemporal imprecision remain central barriers that emerging delivery platforms aim to address. This synthesis is offered as a resource for investigators designing mechanistic and translational studies, rather than as a prescriptive therapeutic framework.

##  Supplemental Information

10.7717/peerj.21480/supp-1 Supplemental Information 1 Graphical Abstract Schematically illustrates the core crosstalk between MDM2/Notch signaling pathway and ferroptosis, and summarizes its multifaceted regulatory roles in cancer metabolic rewiring, immune evasion, and organ-specific metastasis.

10.7717/peerj.21480/supp-2 Supplemental Information 2 Highlights of the work

## Additional Information and Declarations

### Data Availability

The following information was supplied regarding data availability: This is a literature review.
