# Supplementary figures and images for: MDM2/Notch-Ferroptosis crosstalk in cancer: metabolic rewiring, immune evasion, and organ-specific metastasis
Source: PeerJ. 2026 Jun 25;14:e21480. doi: 10.7717/peerj.21480 (PMC13310486; doi:10.7717/peerj.21480)

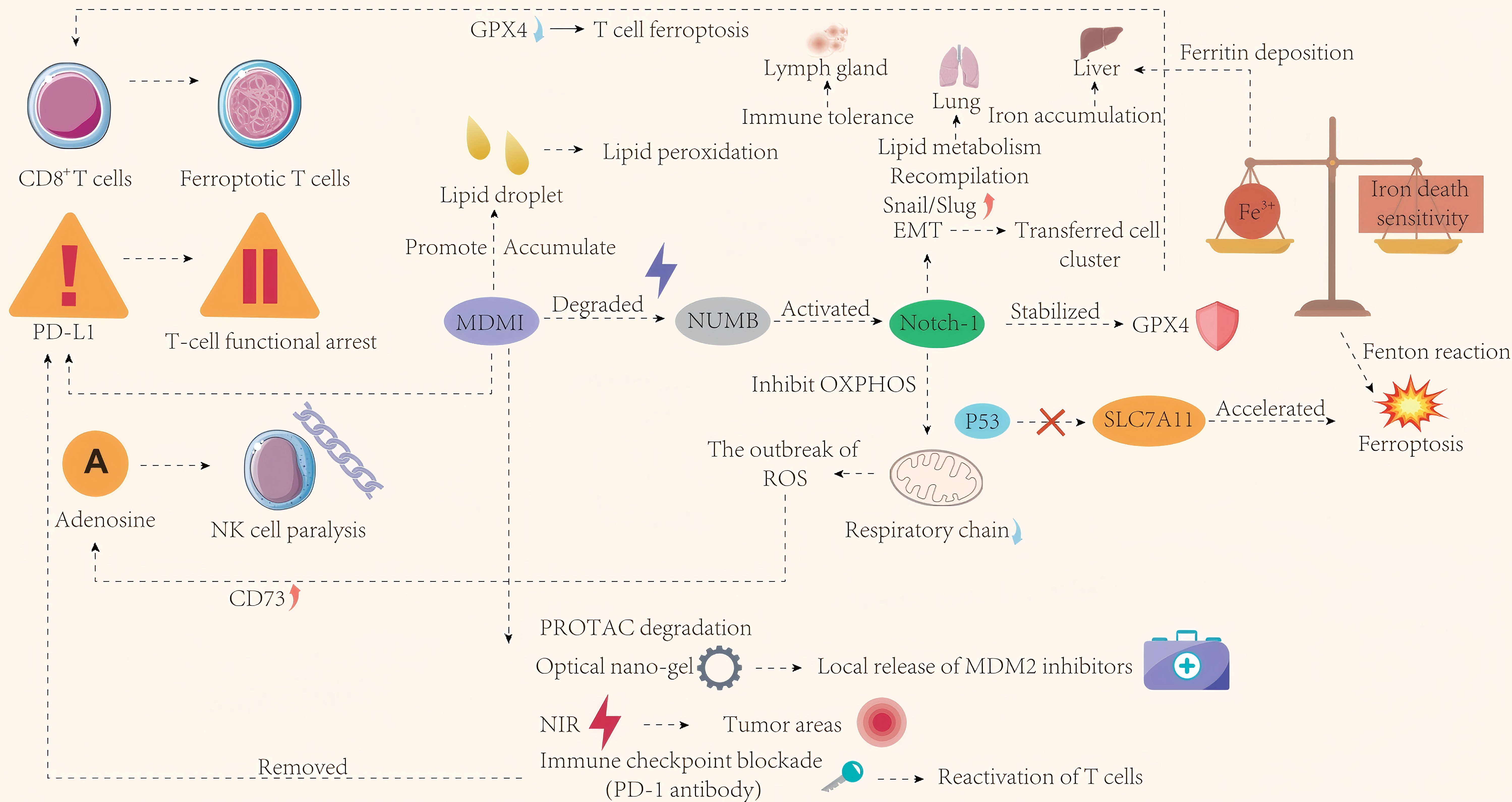

Supplement: Supplemental Information 1 — Schematically illustrates the core crosstalk between MDM2/Notch signaling pathway and ferroptosis, and summarizes its multifaceted regulatory roles in cancer metabolic rewiring, immune evasion, and organ-specific metastasis. [file peerj-14-21480-s001.png]
