# Supplementary material for: MDM2/Notch-Ferroptosis crosstalk in cancer: metabolic rewiring, immune evasion, and organ-specific metastasis
Source: PeerJ. 2026 Jun 25;14:e21480. doi: 10.7717/peerj.21480 (PMC13310486; doi:10.7717/peerj.21480)
Supplement: Supplemental Information 2 [file peerj-14-21480-s002.docx]

Highlights

This review introduces the novel concept of the "MDM2/Notch-ferroptosis axis" as a unified driver of tumor progression.

The axis orchestrates a tri-dimensional network linking metabolic reprogramming, immune evasion, and organ-specific metastasis.

MDM2 and Notch converge on ferroptosis guardians (GPX4, p53, SLC7A11) via ubiquitin-proteasome and transcriptional control.

Metabolic rewiring driven by the axis shapes an immunosuppressive microenvironment and promotes organotropic colonization.

We propose spatiotemporally-resolved therapeutic strategies to overcome paradoxes and synergize ferroptosis induction with immunotherapy.
